# Retrospective Analysis of Dosimetric Comparison Between Intensity-Modulated Radiation Therapy and Volumetric-Modulated Arc Therapy in Patients With Esophageal Cancer

**DOI:** 10.7759/cureus.76981

**Published:** 2025-01-05

**Authors:** Vishwadeep Mishra, Rashmi Yadav, Shwetima Chaudhary, Laxman Pandey, Archana Pandey

**Affiliations:** 1 Radiation Oncology, All India Institute of Medical Sciences, Gorakhpur, IND; 2 Radiation Oncology, Hind Institute of Medical Sciences, Barabanki, IND; 3 Radiation Oncology, TS Mishra Medical College, Lucknow, IND; 4 Radiation Oncology, Rohilkhand Medical College and Hospital, Bareilly, IND

**Keywords:** esophageal cancer, intensity-modulated radiation therapy, organs at risk, radiotherapy, volumetric arc therapy

## Abstract

Introduction

Esophageal cancer is a significant global health concern, with high incidence and mortality rates, particularly in India, where it ranks among the top causes of cancer-related deaths. Radiotherapy plays a critical role in the treatment of advanced-stage esophageal cancer. This study aims to compare the dosimetric outcomes of intensity-modulated radiotherapy (IMRT) and volumetric-modulated arc therapy (VMAT) to evaluate their efficacy and safety in managing mid-esophageal carcinoma.

Materials and methods

A retrospective study was carried out on thirty patients with middle-third esophageal cancer who received treatment at Rohilkhand Medical College and Hospital, Bareilly, India. The patients, aged between 50 and 70 years (mean age of 66.5 years), were in stages II to III of cancer according to the American Joint Committee on Cancer (AJCC) 2018 guidelines. All patients had histologically confirmed cases of moderately differentiated squamous cell carcinoma. The treatment procedure included immobilization using a thoracic mold, CT simulation with intravenous and oral contrast, and contouring of the gross tumor volume (GTV), clinical target volume (CTV), planning target volume (PTV), and organs at risk (OARs) following the Radiation Therapy Oncology Group (RTOG) 0436 protocol. VMAT planning was done using the Varian Eclipse™ Treatment Planning System, while IMRT planning employed a seven-field non-coplanar beam setup. Comparative virtual IMRT plans were generated for these patients. Both VMAT and IMRT plans were evaluated based on dosimetric parameters for the PTV and OARs.

Results

Both VMAT and IMRT achieved sufficient PTV coverage, with no statistically significant differences in dosimetric parameters (dose to 99 % volume of PTV, dose to 95 %volume of PTV, maximum dose to PTV, minimum dose to PTV). VMAT demonstrated reduced lung and heart doses compared to IMRT; however, the observed differences were not statistically significant. There was a reduction in lung dose with VMAT when evaluating the dose-volume constraints: volume receiving 15 Gy dose (V15) by 11%, volume receiving 20 Gy dose (V20) by 20%, and volume receiving 25 Gy dose (V25) by 41%, though these differences were not statistically significant. The mean maximum spinal cord dose was significantly lower with VMAT (19.69 Gy) compared to IMRT (30.80 Gy, p=0.01). Heart dosimetry showed slight improvements with VMAT, particularly in volume receiving 30 Gy dose (V30), volume receiving 40 Gy dose (V40), and mean heart dose, though these differences were not statistically significant.

Conclusion

Both VMAT and IMRT provided similar PTV coverage. VMAT showed a reduction in spinal cord dose, which was statistically significant, and a trend toward lower lung and heart doses, though these differences were not statistically significant. VMAT appears to be an effective option for treating mid-esophageal carcinoma while reducing exposure to critical organs.

## Introduction

In 2022, esophageal cancer (EC) accounted for an estimated 511,054 new cases and approximately 445,391 deaths globally, highlighting its significant burden as a major cause of cancer-related mortality worldwide [1]. EC occupies the sixth position in India in terms of incidence (4.9% of all cancers) and mortality (5.9% of all cancers) for 2018. The absolute number of EC cases for 2018 was 52,396, and the number of deaths was 46,504 [2]. Radiotherapy is crucial in the treatment of esophageal carcinoma, particularly as more than 60% of patients are diagnosed at advanced stages where surgery is not an option. Additionally, radiotherapy plays an equally important role in the neoadjuvant setting. Traditionally, radiotherapy for esophageal cancer has utilized various beam arrangements, such as the anteroposterior/posteroanterior (AP/PA) field arrangement, the four-field box technique, and the three-field technique and often forcing a choice between covering the tumor fully and protecting healthy tissues. This can lead to more side effects, treatment delays, and lower success rates. Newer methods like intensity-modulated radiotherapy (IMRT) and volumetric-modulated arc therapy (VMAT) solve these issues by targeting the tumor more precisely while protecting nearby organs [3]. However, recent advancements in treatment planning and delivery have shifted towards more innovative technologies [4-9].

VMAT is an advanced form of IMRT, first introduced by Yu in 1995. VMAT allows for intensity-modulated radiation to be delivered during gantry rotation, incorporating dynamic multi-leaf collimator (MLC) motion, variable dose rates, and gantry speed modulation. This dynamic modulation facilitates highly conformal dose distributions while also reducing treatment time. The shorter treatment duration with VMAT decreases the risk of patient movement during treatment, thereby minimizing the potential for missing the planning target volume (PTV). Compared to static IMRT, VMAT provides similar or better dosimetry within a significantly reduced delivery time [10]. Previous studies have shown its effectiveness in treating various cancers, including head and neck cancer, prostate cancer, and lung cancer [11-13]. We conducted a retrospective study comparing VMAT with IMRT in the treatment of esophageal cancer. This study focused on evaluating dosimetric parameters for the planning target volume (PTV) and organs at risk (OAR).

## Materials and methods

This study retrospectively examined patients with middle-third esophageal cancer who were treated at Rohilkhand Medical College and Hospital, Bareilly, India. All patients were diagnosed at stages II or III, according to the American Joint Committee on Cancer (AJCC) cancer staging manual eighth edition, and received radical treatment using the VMAT technique between July and December of 2023. This analysis aimed to compare dosimetric parameters between VMAT and IMRT techniques. For each patient, an IMRT plan was created for theoretical comparison, resulting in 30 VMAT (group I) and 30 theoretical IMRT (group II) treatment plans for analysis.

Inclusion and exclusion criteria

This study included patients aged 18 to 70 years diagnosed with middle-third esophageal cancer and squamous cell carcinoma. Patients with a history of prior surgery or radiotherapy, as well as those with other metastatic conditions, were excluded from the analysis.

Radiotherapy planning

Immobilization and Simulation

Patients were immobilized in a supine position using a uniform thermoplastic thoracic mold with arms positioned above the head. The simulation was performed using IV and oral contrast-enhanced CT imaging with a slice thickness of 2.5 mm, covering from the C2 to the L4 vertebrae.

Delineation of PTV and Organs at Risk

Contouring was done for the gross tumor volume (GTV), clinical target volume (CTV), planning target volume (PTV), and critical organs, including the lung, heart, and spinal cord following the Radiation Therapy Oncology Group (RTOG) 0436 protocol. The GTV was defined by visible tumors or involved lymph nodes on CT, often incorporating positron emission tomography (PET)-CT fusion for accuracy. The CTV included a 3 cm superior-inferior margin and a 1.5 cm radial margin around the GTV. Lymph nodes were contoured with a uniform 1 cm margin in all directions while considering anatomical landmarks like the lungs and bones. An additional 0.5 cm margin was added to the CTV for the PTV. The heart and left ventricle were contoured in line with the cardiac contouring atlas, and the left anterior descending artery (LAD) was delineated with the pericardial sac and anatomical reference points. PTV dose objectives were specified to be within 95-107% of the prescription dose.

VMAT Planning

VMAT plans were done previously using the Varian Eclipse™ Treatment Planning System (Version 17.00), utilizing two full opposing arcs with 177 control points for optimal target coverage. An anisotropic analytical algorithm with a 2.5 mm grid size was used for dose calculations. Consistent planning objectives were applied across VMAT and IMRT plans to ensure PTV coverage while minimizing dose to critical organs, including the heart and LAD. 

IMRT Planning

**Figure 1:**
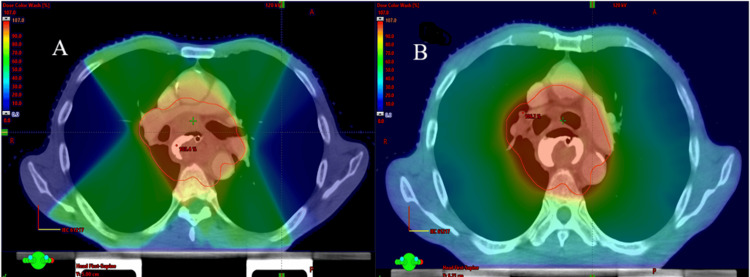
Color dose wash for 0% to 107% range IMRT (A) and VMAT (B) plans, respectively A: IMRT plan with multiple coplanar beam arrangements with different gantry angles; B: VMAT plan showing two full opposing arcs with multiple (177) control points for optimal target coverage

IMRT plans were created virtually based on the same CT imaging and contouring used for the VMAT plans, with optimization focused on covering at least 95% of the PTV with 95% of the prescribed dose and minimizing exposure to OARs. Eclipse™ Treatment Planning System was used for inverse planning with 100 optimization iterations and semi-automatic segmentation. A seven-field coplanar arrangement was used, with gantry angles at 0°, 51°, 102°, 153°, 204°, 255°, and 306°, and consistent isocenter placement. Normal tissue objectives were included in the optimization for IMRT, similar to VMAT. Figures 1 A and B show planning color dose wash for IMRT and VMAT.

Dose Prescription

Treatment was delivered in two phases: phase I included 45 Gy in 25 fractions at 1.8 Gy per fraction, followed by a phase II boost of 5.4 Gy in three fractions at 1.8 Gy per fraction.

Plan Evaluation

Dose-volume histograms were used to evaluate all treatment plans. For the PTV, the parameters analyzed included dose to 99 % volume of PTV (D99%), dose to 95 %volume of PTV (D95%), maximum dose, and minimum dose. Dosimetry for the heart and LAD was assessed in terms of volume receiving 5 Gy dose (V5), volume receiving 10 Gy dose (V10), volume receiving 20 Gy dose (V20), volume receiving 40 Gy dose (V40), and volume receiving 50 Gy dose (V50) as well as minimum (Dmin) and maximum dose (Dmax).

Statistical analysis 

Two-tailed paired t-tests were performed to determine statistical significance, with a threshold of p<0.05. Statistical analyses were conducted using SPSS for Windows, Version 16.0 (SPSS Inc., Chicago, USA).

## Results

This study retrospectively examined patients with middle-third esophageal cancer who were treated at Rohilkhand Medical College and Hospital, Bareilly, India, between July and December 2023. The patient cohort, comprising 30 individuals, included 21 males and nine females. The age range of the cohort was 50-70 years, with a mean age of 66.5 years. Histopathologically, about 60% of patients (18 out of 30) had moderately differentiated squamous cell carcinoma, while the remaining cases consisted of well-differentiated squamous cell carcinoma. In dosimetric results, the PTV coverage was similar between both arms. Dosimetric details for PTV coverage are shown in Table 1.

**Table 1:** Mean dosimetric parameters for ensuring PTV coverage in both groups D99 - dose to 99% volume of PTV; D95 - dose to 95% volume of PTV; Dmax - maximum dose to PTV; Dmin - minimum dose to PTV; VMAT - volumetric-modulated arc therapy; IMRT - intensity-modulated radiotherapy; PTV - planning target volume

Dosimetric parameters (PTV)	VMAT (group I)	IMRT (group II)	p-value	t-value
D99 (Gy)	45.73	44.32	0.73	0.17
D95 (Gy)	47.92	46.87	0.38	0.1
Dmax (Gy)	52.12	51.27	0.42	0.6
Dmin (Gy)	43.64	42.84	0.56	0.96

**Figure 2:**
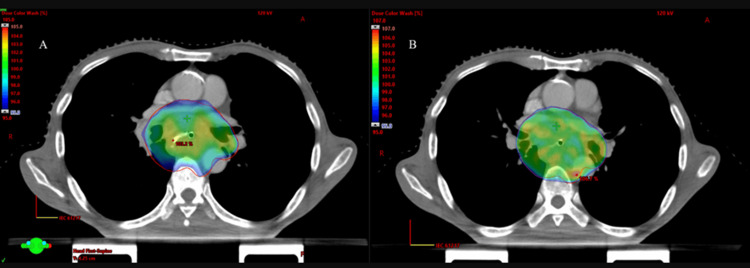
Color dose distribution in the axial section 95% to 107% IMRT (A) and VMAT (B), respectively VMAT - volumetric-modulated arc therapy; IMRT - intensity-modulated radiotherapy

**Figure 3:**
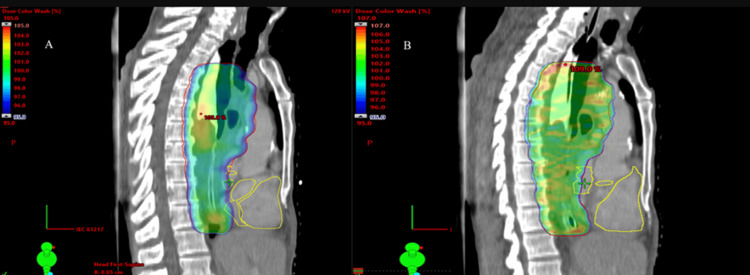
Color dose distribution in the sagittal section IMRT (A) and VMAT (B) VMAT - volumetric-modulated arc therapy; IMRT - intensity-modulated radiotherapy

Both techniques provided adequate coverage of the PTV, ensuring that 95% of the prescription dose was delivered to 100% of the PTV volume. With a prescription dose of 50.4 Gy to the PTV, the dosimetric results (Dmax, Dmin, D99, D95) for all thirty patients using both techniques are summarized in Table 1. The mean values for Dmin, Dmax, D99, and D95 for the PTV in the IMRT and VMAT plans were as follows: Dmin 42.84 Gy vs. 43.64 Gy, Dmax 51.27 Gy vs. 52.12 Gy, D99 44.32 Gy vs. 45.73 Gy, and D95 46.87 Gy vs. 47.92 Gy, respectively. On average, the D99 and D95 values were approximately 1% higher for the VMAT plans compared to the VMAT plans, but this difference was not statistically significant. Dosimetric results showed that the PTV coverage was similar between VMAT group I and IMRT group II (see Figures 2-3).

The reduction in lung volume receiving higher doses (V15, V20, and V25) with VMAT compared to IMRT is a clear benefit for lung tissue preservation, potentially reducing the risk of radiation-induced lung damage. The percentages of lung volume receiving these doses were reduced in the VMAT plans compared to the IMRT plans: V15 was 40.72% vs. 45.32%, V20 was 18.14% vs. 26.46%, V25 was 11.50% vs. 19.46%, V5 was 65.51% vs. 70.33%, and V10 was 67.72% vs. 59.30%; the differences were less consistent, and all comparisons lacked statistical significance. The mean lung dose (MLD) was lower with the VMAT technique compared to IMRT, being 13.85 Gy versus 14.87 Gy. While there was no significant difference in heart dose overall, VMAT showed better sparing for higher doses (V30, V40, and V50), suggesting an advantage in minimizing radiation exposure to critical cardiac structures during esophageal cancer treatment. However, IMRT performed better for V10 (84.73% vs. 77.42%) and V5 (85.79% vs. 80.26%), although these differences were not statistically significant. The mean heart dose was lower with VMAT at 15.86 Gy compared to 20.68 Gy with IMRT. Additionally, the significant reduction in the maximum spinal cord dose (Dmax) with VMAT (19.69 Gy vs. 30.80 Gy for IMRT) is crucial for preventing radiation-induced damage to the spinal cord, highlighting the superior precision of VMAT in protecting surrounding critical organs. Table 2 provides the comparative dosimetric data for the heart, lung, and spinal cord in both treatment arms, highlighting the dosimetric advantages of each technique.

**Table 2:** Mean dosimetric parameters for heart, lung and spinal cord in both groups Paired t-test was used to calculate p-values. V5: volume receiving 5 Gy dose;  V10 - volume receiving 10 Gy dose; V20 - volume receiving 20 Gy dose; V25 - volume receiving 25 Gy dose; V30 - volume receiving 30 Gy dose; V40 - volume receiving 40 Gy dose; V50 - volume receiving 50 Gy dose; Dmax - maximum dose to the spinal cord; VMAT - volumetric-modulated arc therapy; IMRT - intensity-modulated radiotherapy

Organ	Dosimetric parameters	VMAT	IMRT	t-value	p-value
Heart	V5 (%)	85.51	80.26	0.5	0.5
	V10 (%)	84.73	77.42	0.37	0.36
	V30 (%)	18.56	22.14	0.44	0.22
	V40 (%)	8.45	11.11	0.1	0.09
	V50 (%)	2.94	3.03	0.91	0.98
	Mean (Gy)	15.86	20.68	0.02	0.65
Lung	V5 (%)	65.51	70.33	0.11	0.1
	V10 (%)	67.72	59.30	0.34	0.34
	V15(%)	40.72	45.32	0.52	0.5
	V20 (%)	18.14	26.46	0.15	0.21
	V25 (%)	11.50	19.46	0.11	0.11
	Mean (Gy)	13.85	14.87	0.97	0.97
Spinal cord	Dmax (Gy)	19.69	30.80	0.01	0.01

## Discussion

In this study, we compared the dosimetric parameters of two plans of patients with esophageal carcinoma treated with VMAT and IMRT. A study by Yin et al. effectively highlights the significant advantage of VMAT in reducing treatment times by 50% to 70% compared to static IMRT, which not only improves patient convenience but also minimizes the potential risks associated with prolonged radiation exposure [14]. This technique has gained popularity due to its efficiency and effectiveness in radiation delivery. Previous studies have demonstrated that VMAT achieves comparable or superior target conformity and homogeneity, with minor increases in low-dose radiation exposure to organs at risk, like the lungs and heart, while reducing high-dose exposure [15]. The study's findings that VMAT achieved nearly identical PTV homogeneity and OAR sparing to IMRT while demonstrating superior lung sparing (especially in V20 and V30) and better heart dose distribution emphasize the potential of VMAT to optimize treatment effectiveness while minimizing harm to surrounding critical tissues. Research by Van Benthuysen et al. found that both IMRT and VMAT were effective in delivering 95% of the prescribed dose to the entire PTV volume in most cases, except for one instance with VMAT where the minimum dose to the PTV was 90% [12]. Similarly, a study by Yin et al. revealed that VMAT provided superior lung sparing compared to IMRT, particularly regarding V20 and V30. VMAT also demonstrated lower percentages of V30, V40, and V50 for the heart [14]. In our study, VMAT resulted in reduced lung doses compared to IMRT, with reductions of 4.6%, 8.32%, 7.9%, and 1.02% for V15, V20, V25, and mean dose, respectively. For the heart, VMAT achieved modest dose reductions compared to IMRT, with decreases of 3.56%, 2.66%, 0.09%, and 4.82% for V30, V40, V50, and mean dose, respectively. Wei et al. reported that maintaining the V30 of the heart below 46% resulted in 13% of patients exhibiting pericardial effusion [16]. In our investigation, the mean V30 for the heart remained below 23% for both treatment methods, with VMAT showing superior heart dose reduction compared to IMRT, having V30 values of 22.14% versus 18.56% (p=0.38). Hawkins et al. examined VMAT's efficacy in minimizing heart and spinal cord exposure while keeping lung V20 below 20% for lower gastroesophageal tumors. Their comparison of IMRT (four-field) and VMAT plans revealed that VMAT significantly reduced heart V30 (31% vs. 55%) and achieved a better Conformity Index (CI) in a shorter treatment time [17].

Additionally, Van Benthuysen et al. found that the maximum dose delivered to the spinal cord was only 2.12 Gy higher with VMAT, a difference considered clinically insignificant [12]. In our investigation, a significant disparity was observed between the two techniques regarding maximum spinal cord dose, with Dmax values of 30.80 Gy for IMRT and 19.69 Gy for VMAT (p=0.01). Studies like those by Zhang et al. and Yamashita et al. further demonstrate VMAT's enhanced conformity and homogeneity over IMRT in cases requiring high precision, such as esophageal and lung cancers [18,13]. Their findings corroborate our results, showing VMAT's capacity to spare nearby organs while maintaining PTV coverage and heart doses. Consistent with our findings, Kataria et al. reported that VMAT reduced the volume of lung tissue receiving moderate to high doses (V20, V30) compared to IMRT [19]. This reduction is significant in reducing pulmonary toxicity risk in patients with esophageal carcinoma. Similarly, in a study by Guo et al., VMAT was found to limit doses to the heart and lung without compromising tumor control, supporting our observation of reduced mean lung dose and better heart sparing [20]. This aligns with our findings and emphasizes VMAT's potential to reduce pulmonary and cardiac toxicity risk in the treatment of mid-esophageal carcinoma. A study by Lin et al. noted a significant treatment time reduction in VMAT plans for esophageal cancer, resulting in better patient throughput and less chance of patient movement during treatment sessions, thus improving overall treatment accuracy [11]. Proton therapy is an alternative to photon-based VMAT and IMRT, and new evidence by Karube et al. suggests that proton therapy offers superior dose distributions with even lower OAR exposure than VMAT [21]. Although still experimental, proton therapy might further reduce cardiopulmonary doses in esophageal cancer treatment, indicating a potential shift in future treatment protocols.

This study has some limitations. Its retrospective nature may introduce biases due to incomplete or inconsistent documentation of treatment plans and outcomes. The relatively small sample size may limit the generalizability of the results to a broader population. Additionally, the study was conducted at a single institution, which may not fully reflect practices and outcomes from diverse clinical settings. The population was limited to patients with mid-esophageal carcinoma, potentially excluding the variability seen in patients with different tumor locations or stages. Lastly, the study primarily focused on technical aspects of radiation delivery, overlooking important measures such as patient-reported outcomes and quality of life.

## Conclusions

Our research highlights the advantages of VMAT over IMRT in treating mid-esophageal carcinoma. Both techniques ensured adequate PTV coverage, delivering the prescribed dose effectively. While VMAT demonstrated superior organ sparing, the statistically significant difference was observed specifically in the maximum dose reduction to the spinal cord. Although differences in mean doses and volumes receiving high doses in critical organs like the lungs and heart were noted, these did not reach statistical significance. These findings support the role of VMAT as a safe and efficient modality in esophageal cancer management, and further studies with a focus on treatment efficiency and broader patient populations are encouraged to validate these results.

## References

[1] Teng Y, Xia C, Cao M (2024). Esophageal cancer global burden profiles, trends, and contributors. Cancer Biol Med.

[2] Bray F, Ferlay J, Soerjomataram I, Siegel RL, Torre LA, Jemal A (2018). Global cancer statistics 2018: GLOBOCAN estimates of incidence and mortality worldwide for 36 cancers in 185 countries. CA Cancer J Clin.

[3] Bradley JD, Muti S (2012). Carcinoma of the esophagus. Technical Basis of Radiation Therapy: Practical Clinical Applications. 5. ed.

[4] Fenkell L, Kaminsky I, Breen S, Huang S, Van Prooijen M, Ringash J (2008). Dosimetric comparison of IMRT vs. 3D conformal radiotherapy in the treatment of cancer of the cervical esophagus. Radiother Oncol.

[5] Choi KH, Kim J, Lee SW, Kang YN, Jang H (2018). Dosimetric comparison between modulated arc therapy and static intensity modulated radiotherapy in thoracic esophageal cancer: a single institutional experience. Radiat Oncol J.

[6] Chandra A, Guerrero TM, Liu HH (2005). Feasibility of using intensity-modulated radiotherapy to improve lung sparing in treatment planning for distal esophageal cancer. Radiother Oncol.

[7] Wang SL, Liao Z, Liu H, Ajani J, Swisher S, Cox JD, Komaki R (2006). Intensity-modulated radiation therapy with concurrent chemotherapy for locally advanced cervical and upper thoracic esophageal cancer. World J Gastroenterol.

[8] Vosmik M, Petera J, Sirak I (2010). Technological advances in radiotherapy for esophageal cancer. World J Gastroenterol.

[9] Wong JW, Sharpe MB, Jaffray DA (1999). The use of active breathing control (ABC) to reduce margin for breathing motion. Int J Radiat Oncol Biol Phys.

[10] Yu CX (1995). Intensity-modulated arc therapy with dynamic multileaf collimation: an alternative to tomotherapy. Phys Med Biol.

[11] Lin CY, Huang WY, Jen YM, Chen CM, Su YF, Chao HL, Lin CS (2014). Dosimetric and efficiency comparison of high-dose radiotherapy for esophageal cancer: volumetric modulated arc therapy versus fixed-field intensity-modulated radiotherapy. Dis Esophagus.

[12] Van Benthuysen L, Hales L, Podgorsak MB (2011). Volumetric modulated arc therapy vs. IMRT for the treatment of distal esophageal cancer. Med Dosim.

[13] Yamashita H, Haga A, Takahashi W, Takenaka R, Imae T, Takenaka S, Nakagawa K (2014). Volumetric modulated arc therapy for lung stereotactic radiation therapy can achieve high local control rates. Radiat Oncol.

[14] Yin L, Wu H, Gong J (2012). Volumetric-modulated arc therapy vs. c-IMRT in esophageal cancer: a treatment planning comparison. World J Gastroenterol.

[15] Xu C, Xi M, Komaki R (2017). Dosimetric and clinical outcomes after volumetric modulated arc therapy for carcinoma of the thoracic esophagus. Adv Radiat Oncol.

[16] Wei X, Liu HH, Tucker SL (2008). Risk factors for pericardial effusion in inoperable esophageal cancer patients treated with definitive chemoradiation therapy. Int J Radiat Oncol Biol Phys.

[17] Hawkins MA, Bedford JL, Warrington AP, Tait DM (2012). Volumetric modulated arc therapy planning for distal oesophageal malignancies. Br J Radiol.

[18] Zhang WZ, Zhai TT, Lu JY, Chen JZ, Chen ZJ, Li DR, Chen CZ (2015). Volumetric modulated arc therapy vs. c-IMRT for the treatment of upper thoracic esophageal cancer. PLoS One.

[19] Kataria T, Govardhan HB, Gupta D (2014). Dosimetric comparison between volumetric modulated arc therapy (VMAT) vs intensity modulated radiation therapy (IMRT) for radiotherapy of mid esophageal carcinoma. J Cancer Res Ther.

[20] Guo XQ, Mao RH, Liu B, Ge H (2023). Study on esophageal cancer radiotherapy dosimetry and position verification for volumetric modulated arc therapy. Asian J Surg.

[21] Karube M, Nakayama H (2021). Proton therapy for patients with esophageal cancer: History, characteristics, clinical outcome and future direction of proton beam therapy. Glob Health Med.

